# Evaluation of the impact of postural restrictions after Semont liberatory maneuver on immediate reactions and short-term outcome in the posterior semicircular canal canalolithiasis: a preliminary study

**DOI:** 10.3389/fneur.2025.1497156

**Published:** 2025-02-10

**Authors:** Andrea Albera, Marco Boldreghini, Sergio Lucisano, Roberto Albera, Claudia Cassandro, Alma Barci, Giuseppe Riva, Andrea Canale

**Affiliations:** Department of Surgical Sciences, University of Turin, Turin, Italy

**Keywords:** residual dizziness, liberating nystagmus, postural restrictions, orthotropic nystagmus, Semont maneuver

## Abstract

**Introduction:**

Benign paroxysmal positional vertigo is the most frequent peripheral vestibular disorder, characterized by brief but intense vertigo crises related to changes in position. The Liberatory maneuver is considered the gold-standard treatment, with a short-term resolution of the vertigo in over 70% of cases, and achieving a 90% success rate after four maneuvers. An immediate reaction to the subsequent repositioning maneuver is often an orthotropic nystagmus (Ny) occurring in the same direction as the Ny observed after returning to the primary position. This reaction, occurring seconds to minutes after reaching the second position, is considered a positive predictor of the maneuver’s effectiveness. To improve the success rate after the maneuver, many authors have suggested postural restrictions.

**Methods:**

To determine the best predictors of outcome between the immediate behavior after the Semont maneuver and postural restriction, we analyzed 102 patients with posterior semicircular canalithiasis who underwent the Semont maneuver. In each case, we assessed the immediate reaction to the maneuver. Postural restrictions were recommended to 55 participants, while the remaining 40 were instructed to engage in normal head movements, even immediately following the maneuver.

**Results:**

The resolution rate was almost the same (69% versus 62%), regardless of postural behavior, while a significantly high success rate was obtained in the presence of Ny in position 2 of the Semont maneuver.

**Conclusion:**

Our results support the hypothesis that postural restriction is not necessary after the Semont maneuver and that the occurrence of Ny during position 2 is the main outcome indicator.

## 1 Introduction

Benign paroxysmal positional vertigo (BPPV) is the most frequent vestibular disorder and is characterized by recurrent acute vertigo crises triggered by the assumption of certain positions such as lying down, getting up, and rolling over in the bed (1). The disease was first described by Barany and was named by Dix and Hallpike who also described a diagnostic test (2). In 60–90% of cases, BPPV affects the posterior semicircular canal; the lateral canal is occasionally involved and the superior canal is rarely the cause of the disorder (3). Canalolithiasis is a widely accepted theory to explain the disease by suggesting that free-floating particles move along the canal under the influence of gravity during head movements into a provoking position. This movement creates a hydrodynamic pull in the endolymph that deflects the cupola, leading to vertigo and nystagmus (Ny) (4).

The Liberatory maneuver of Semont (LM) is considered the gold-standard treatment of BPPV, with a short-term resolution of vertigo in more than 70% of cases, and a success rate of 90% after four maneuvers (5). The LM acts by moving the particles from the involved canal to the utricle. For cases involving the posterior canal, Semont’s (SM) and Eply’s canalith repositioning procedure maneuvers are considered the most effective therapeutic procedures (6, 7). The principal side effects after LM in cases of posterior canal BPPV are the conversion to lateral canal BPPV (8), persistent postural unsteadiness, and residual dizziness (9). Immediate recurrence is rarely reported, while long-term recurrence is described in approximately 50% of the cases (2).

A typical immediate reaction to repositioning maneuvers is the appearance of an orthotropic Ny, in the same direction as the Ny, reported after reaching the primary position. This reaction is associated with paroxysmal vertigo that appears when the patient reaches the position with the head turned toward the safe ear (the second position). The appearance of this intense reaction several seconds to minutes after reaching the second position is defined as liberatory vertigo and is considered a positive predictive factor for maneuver efficacy in a single case (10).

In the case of immediate failure of the liberatory maneuver, it has been hypothesized that the free-floating particles do not reach the utricle. However, another possibility is that, after reaching the utricle, the particles may return to the canal following head movements performed in the days after the maneuver. Therefore, some authors have suggested avoiding head movements for several days after liberatory therapy (1).

To predict an improvement in the success rate of LM for posterior canal canalolithiasis, this study aimed to evaluate the immediate reaction to SM, the efficacy of avoiding head movements after LM, and evaluate which of the two can be more relevant to the immediate outcome.

## 2 Materials and methods

This study included 102 participants affected by BPPV due to unilateral long-arm posterior canalolithiasis with geotropic Ny. We excluded patients affected by:

- canalothiasis due to horizontal or superior canal;
- canalolithiasis-inducing apogeotropic Ny;
- multiple canal involvement;
- deficit of vestibular function at the Video Head Impulse Test (VHIT);
- comorbidity that could make carrying out the SM difficult;
- duration of the acute phase less than 2 weeks;

Patients already treated with liberatory maneuvers in the same acute phase were also excluded from the study. All patients underwent accurate anamnesis, otoscopy, and an assessment of the spontaneous, positional, and positioning Ny using the Dix-Hallpike (DH), Pagnini-McClure maneuvers, and VHIT. Their ocular movements were evaluated using video oculoscopy.

As mentioned previously, participants with an abnormal VHIT or other functional modifications were excluded from the study. After the diagnosis, all patients underwent SM. During the maneuver, we evaluated the appearance of vertigo and orthotropic Ny after reaching the second position (safe ear down), the appearance of vertigo and Ny in the third position (returning to the sitting position), and the absence of reaction (vertigo or Ny). The immediate outcome of repeating the DH maneuver was not analyzed in any case.

Based on a randomized protocol, 62 patients (59%) were instructed to avoid lying on the affected ear and to perform head movements along the roll (right and left) and pitch axes (up and down) while standing (group A). For the remaining 40 patients (41%) in group B, we instructed them to perform normal head movements immediately post-maneuver. As many patients avoid spontaneously lying in a free position and perform roll and pitch head movements after LM (11), we strongly suggested that they assume the preferred position when sleeping and perform normal head movements when standing.

All patients admitted to the study were re-examined 6–9 days after the SM (range 7, 9 days). Seven of the 62 participants included in group A were excluded from the study as they did not observe the head movement restrictions. Therefore, the overall sample was composed of 95 patients: 35 males (37%) and 60 females (63%), with a mean age of 67 years (range 34–89) meanwhile, group A was composed of 55 patients.

Group A comprised 25 males (45%) and 30 females (55%), with a mean age of 66 (SD 12). Group B comprised 15 males (38%) and 25 females (62%), with a mean age of 68 years (SD 13). Sex distribution and age did not differ significantly between the groups (p > 0.05, chi-squared test, and Student’s t-test).

The control visit after SM was always performed by the same examiner who diagnosed the BPPV, and the examiner was not informed of the group to which the patient was assigned. The evaluation of the outcome was conducted based on patient reports on the absence or presence of positional vertigo and the result of the DH maneuver to improve the success rate of the LM.

BPPV after LM was considered cured if both positioning vertigo and Ny were absent. The BPPV was considered not cured if both or either vertigo/Ny remained, or only improvement was observed (presence of vertigo/Ny of lower intensity).

**Figure 1:**
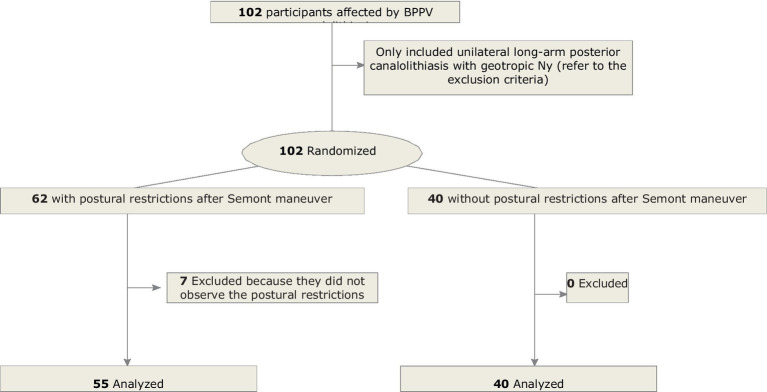
Study flowchart.

The workflow is shown in Figure 1.

Statistical analyses were performed using SPSS, and the level of significance was set at *p* < 0.05. This study was approved by the ethics committee of the AOU CDSS (code 0059821 31/5/2021). All patients provided informed consent for the study submission. The study protocol complied with the recommendations of the Declarations of Helsinki and Tokyo.

## 3 Results

In 67 of 95 (65%) patients in the control group, BPPV was cured after SM. The resolution of the acute form of BPPV was observed in 69% of patients in group A (postural restriction) and 62% of patients in group B (without postural restriction). The results are depicted in Table 1. The differences in the chi-square test (*p* > 0.05) were not significant.

**Table 1:** Number and rate of the outcome of the SM concerning the postural restriction after SM.

Postural restriction after liberatory maneuver	BPPV cured	BPPV not cured
Yes (55 cases)	38 (69%)	17 (71%)
No (40 cases)	25 (62%)	15 (38%)

Differences were not significant according to the chi-square test (*p* > 0.05).

Considering the results based on the immediate reaction to SM, we observed the least favorable result in participants who presented with vertigo/Ny returning to the sitting position. The results are displayed in Table 2. Differences were considered significant according to the chi-square test (*p* < 0,01).

**Table 2:** Number and rate of the outcome of the liberatory maneuver in relation to the immediate reaction to SM.

Immediate reaction to SM	BPPV cured	BPPV not cured
Vertigo/Ny in SM position 2 (70 cases)	51 (73%)*	19 (27%)*
Vertigo/Ny in SM position 3 (10 cases)	2 (20%)*	8 (80%)*
No vertigo/Ny (15 cases)	10 (67%)*	5 (37%)*

Differences were not significant based on the chi-square test (p < 0.01)*.*p* < 0.01)*.

**Figure 2:**
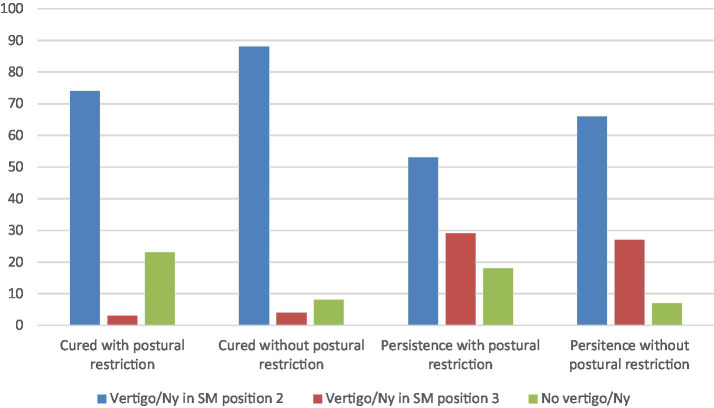
Rate of the outcome of the liberatory maneuver in relation to both the postural restriction and the kind of reaction to the movement. The rate of success is lower in the case of postural restriction and higher in the presence of vertigo/orthotropic nystagmus (Ny) after the Semont maneuver regardless of the participant’s postural behavior. On the other hand, the rate of persistence is higher in the presence of vertigo/Ny in position 3 or in the absence of vertigo/Ny and without postural restriction.

On the basis of data reported in Tables 1, 2 we have evaluated the rate of success or fail in the treatment of BPPV in relation to both postural restrictions and response to SM. Results are reported in Figure 2 that clearly shows a higher rate of success in case of the appearance of vertigo in Semont’s position 2 rather than the application of postural restrictions after therapy, therefore regardless the post-manouver restrictions, the principal parameter related to the outcome seems to be the immediate response to the SM.

Patients who did not show the resolution of BPPV were submitted to a second LM. Results of successive maneuvers are not reported in the paper since this aspect is out of the aim of the study.

## 4 Discussion

BPPV is the most common cause of acute peripheral vertigo (1, 3). BPPV in characterized by brief but intense crisis of acute vertigo consequent to head movements and it is often disabling for patients (3). Moreover, LM is universally accepted as the best therapy for the acute phase, facilitating the removal of the endolymphatic debris (EB) from the canal involved in the majority of cases often with success observed just after the first maneuver. The repositioning of the ED in the utricle allows the patient to be free from positioning vertigo even if, in some cases, it can cause residual dizziness (9).

Given that it is conceivable for the ED to remain in the utricle for a few days, patients are often advised to avoid head movements after the LM that could favor their return to the semicircular canal, thereby favoring the immediate recurrence of BPPV (12). This hypothesis is supported by the statistically significant correlation between the preferred sleep side and the affected side of BPPV, suggesting that the position may facilitate the development of BPPV secondary to otoconia migration into the semicircular canals of the undermost ear due to gravity (13).

Postural restrictions refer to the sleeping position and in previous literature authors suggested laying the patient with several pillows with the head in a near-vertical position (13–16) to avoid moving the head forward or backward when standing (13) or playing sports (13, 16) for a few days, beginning a week after LM (13, 14). The results obtained in previous studies about the effectiveness of postural restrictions are not concordant.

In previous studies, in case of success after the SM, the authors recommended that patients remain in a head-upright position for 24–48 h after the procedure, refrain from lying flat, engage in vigorous physical exercise for at least 24 h, or wear cervical collars for 2 days (11, 17–22).

However, in the series of publications published later, none of the authors confirmed the hypothesis of the utility of this procedure and that this behavior should not be suggested to patients after SM (23–29). Similar results have been reported after EM (26, 30). These conclusions are supported by three meta-analyses (31–33).

Yousovich et al. (23) suggested that after LM, the forced extension of the neck with the head supported and held in the position for 3 min eased the detachment of particles from the cupula and channel wall. This movement could also prevent reverse migration caused by insufficient extension during passage from the first position to the second position, with the authors reporting a 99% success rate.

The usefulness of postural restrictions after LM is in accordance with an experimental model in frogs which demonstrated that otoconia are stably repositioned 3–5 min after a repositioning maneuver (34).

In a relatively recent series of publications, only a few authors affirm (13, 34) the importance of postural restrictions after LM, principally suggesting that the sample sizes in these studies were not sufficient to detect a significant difference and that the effectiveness of postural restriction is limited by the poor compliance of patients. However, they recommend that these restrictions be limited to 2 days.

Our results regarding the correlation between postural restriction and the resolution of the acute phase of posterior canal BPPV strongly support the hypothesis that this procedure is not effective, therefore the risk of immediate reentry of otoconial debris in the canal appears to be low.

Our study was conducted in a relatively large series of cases, and we adopted a blinded procedure for the examiner to evaluate the outcome and avoid any interference in the judgment. As most patients without postural restrictions tend to self-impose a sleep-position restriction (13), which can complicate the evaluation of data, we strongly suggest that patients admitted to group B follow a free postural activity, even including sports, immediately after LM.

In this study, we also evaluated the relationship between the outcome and pattern of response to SM. Consistent with the findings of a previous study on a different series of patients (10), our results confirmed that the appearance of orthotropic Ny and/or vertigo in the second SM position correlates with a significantly high success rate in treating BPPV.

A typical prompt reaction after LMs is the sudden onset of orthotropic Ny coupled with objective vertigo, which manifest in the second position of the SM, namely, the stance with the head turned toward the unaffected ear. This occurrence is considered a positive prognostic sign for the efficacy of the SM (5, 35, 36) since it should confirm the exit of otoconial debris from the posterior semicircular canal to the utricle. However, the absence of such typical orthotropic Ny might suggest that the maneuver did not correctly displace the otoliths toward the utricle. In the present literature, a 70–80% success rate of the SM is described in cases of the onset of an orthotropic Ny in the second position of the maneuver itself. In contrast, in the absence of any clinical objective signs, the success rate drops to 50% (5, 10, 35).

The rate of BPPV resolution was always slightly low in the participants with postural restrictions.

## 5 Limitations

The article has some limitations that might influence its results. First, the sample size (95 actual participants) may not be sufficient to generalize the findings to a larger population, especially considering the variability in individual characteristics of patients with benign paroxysmal positional vertigo (BPPV). In addition, the sample is divided into two groups (with and without postural restrictions), and the possible noncompliance with postural restrictions by some subjects included in Group A may have introduced a behavioral bias that cannot be fully controlled for. Finally, the study protocol did not take into account external factors, such as lifestyle or any comorbidities, that might, in some way, have influenced the outcome. These limitations highlight the need for further studies to confirm or otherwise the observed results.

## 6 Conclusion

In conclusion, our study supports the hypothesis that postural restrictions are not useful in increasing the rate of success after SM and that the principal outcome indicator is the appearance of vertigo/Ny in position 2 SM. This free behavior is well accepted by patients because they are obliged to avoid head movements during illness.

## Data Availability

The raw data supporting the conclusions of this article will be made available by the authors, without undue reservation.

## References

[1] Bhattacharyya N Gubbels SP Schwartz SR Edlow JA El-Kashlan H Fife T. Clinical Practice Guideline: Benign Paroxysmal Positional Vertigo (Update). Otolaryngol Head Neck Surg. (2017) 156:S1–S47. doi: 10.1177/0194599816689667 28248609

[2] Mandalà M Salerni L Nuti D. Benign positional paroxysmal Vertigo treatment: a practical update. Curr Treat Options Neurol. (2019) 21:66. doi: 10.1007/s11940-019-0606-x, PMID: 31807976

[3] von Brevern M Radtke A Lezius F Feldmann M Ziese T Lempert T. Epidemiology of benign paroxysmal positional vertigo: a population based study. J Neurol Neurosurg Psychiatry. (2007) 78:710–5. doi: 10.1136/jnnp.2006.100420, PMID: 17135456 PMC2117684

[4] Parnes LS McClure JA. Free-floating endolymph particles: a new operative finding during posterior semicircular canal occlusion. Laryngoscope. (1992) 102:988–92. doi: 10.1288/00005537-199209000-00006, PMID: 1518363

[5] Mandalà M Santoro GP Asprella Libonati GA Casani AP Faralli M Giannoni B. Double-blind randomized trial on short-term efficacy of the Semont maneuver for the treatment of posterior canal benign paroxysmal position vertigo. J Neurol. (2012) 259:882–5. doi: 10.1007/s00415-011-6272-x, PMID: 22008871

[6] Semont A Freyss G Vitte E. Curing the BPPV with a liberatory maneuver. Adv Otorhinolaryngol. (1998) 42:290–3. doi: 10.1159/000416126 3213745

[7] Epley JM. The canalith repositioning procedure: for treatment of benign paroxysmal positional vertigo. Otolaryngol Head Neck Surg. (1992) 107:399–404. doi: 10.1177/019459989210700310, PMID: 1408225

[8] Scotto di Santillo L Califano L. Canal switch: a possible complication of physical therapeutic manoeuvres for posterior canal benign paroxysmal positional vertigo. Acta Otorhinol Ital. (2023) 43:49–55. doi: 10.14639/0392-100X-N2016, PMID: 36860150 PMC9978300

[9] Casani AP Navari E Albera R Agus G Asprella Libonati G Chiarella G. Approach to residual dizziness after succesfully treated benign paroxysmal positional vertigo: effect of a poliyphenol compound supplementation. Clin Pharmacol. (2019) 11:117–25. doi: 10.2147/CPAA.S210763, PMID: 31534374 PMC6681902

[10] Albera A Boldreghini M Canale A Albera R Gervasio CF. Vertigo returning to the sitting position after tge Semont’s manoeuvre. Is it a prognostic symptom? Acta Otorhinolaryngol Ital. (2018) 38:145–50. doi: 10.14639/0392-100X-1815 29967559 PMC6028822

[11] Wolf JS Boyev KP Manokey BJ Mattox DE. Success of the modified Epley maneuver in treating benign paroxysmal positional vertigo. Laryngoscope. (1999) 109:900–3. doi: 10.1097/00005537-199906000-00011, PMID: 10369279

[12] Babac S Djeric D Petrovic-Lazic M Arsovic N Mikic A. Why do treatment failure and recurrences of benign paroxysmal positional vertigo occur? Otol Neurotol. (2014) 35:1105–10. doi: 10.1097/MAO.0000000000000417, PMID: 24853239

[13] Çakir BO Ercan I Çakir ZA Turgut S. Efficacy of postural restriction in treating benign paroxysmal positional vertigo. Arch Otolaryngol Head Neck Surg. (2006) 132:501–5. doi: 10.1001/archotol.132.5.501, PMID: 16702565

[14] Li S Tian L Han Z Wang J. Impact of postmanoeuvre sleep position on recurrence of benign paroxysmal positional vertigo. PLoS One. (2013):18 e 83566. doi: 10.1371/journal.pone.0083566 PMC3867465 24367602

[15] Oh HJ Kim JS Han BI Lim J. Predicting a successful treatment in posterior canal benign paroxysmal positional vertigo. Neurology. (2007) 68:1219–22. doi: 10.1212/01.wnl.0000259037.76469.e4, PMID: 17420406

[16] Toupet M Ferrary E Bozorg GA. Effect of repositioning menuever type and postmaneuver restrictions on vertigo and dizziness in benign positional paroxysmal vertigo. Sci World J. (2012):162123. doi: 10.1100/2012/162123, PMID: 22973168 PMC3438743

[17] Herdman SJ Tusa RJ Zee DS Proctor LR Mattox DE. Single treatment approaches to benign paroxysmal positional vertigo. Arch Otolaryngol Head Neck Surg. (1993) 11:450–4. doi: 10.1001/archotol.1993.01880160098015 8457308

[18] Harvey Sahain TC Adamiec LC. Modified liberatory maneuver: effective treatment for benign paroxysmal positional vertigo. Laryngoscope. (1994) 104:1206–12. doi: 10.1288/00005537-199410000-00004 7934589

[19] Li JC. Mastoid oscillation: a critical factor for success in the canalith repositioning procedure. Otolaryngol Head Neck Surg. (1995) 112:670–5. doi: 10.1016/S0194-59989570174-5, PMID: 7777350

[20] Epley JM. Caveats in particle repositioning for treatment of canalithiasis (BPPV). Oper Tech Otolaryngol Head Neck Surg. (1997) 8:868–76. doi: 10.1016/S1043-1810(97)80005-X

[21] Nunez RA Cass SP Furman JM. Short- and long-term outcomes of canalith repositioning for benign paroxysmal positional vertigo. Otolaryngol Head Neck Surg. (2000) 122:647–52. doi: 10.1016/S0194-5998(00)70190-2 10793340

[22] Haberkamp TJ Hamid M. The Epley or canalith repositioning maneuvers for classic benign positional vertigo. Oper Tech Otolaryngol Head Neck Surg. (2001) 12:151–6. doi: 10.1016/S1043-1810(01)80011-7

[23] Yousovich R Duvdevani SI Lipschitz N Wolf M Migirov L Yakirevitch A. Correlation between the sleep-position habits and the affected posterior Semicircular Canal in patients with benign paroxysmal positional Vertigo. Isr Med Assoc J. (2019) 21:716–8. PMID: 31713357

[24] Casqueiro JC Ayala A Monedero G. No more postural restrictions in posterior canal benign paroxysmal positional vertigo. Otol Neurotol. (2008) 29:706–9. doi: 10.1097/MAO.0b013e31817d01e8, PMID: 18520622

[25] De Stefano A Dispenza F Citraro L Petrucci AG Di Giovanni P Kulamarva G. Are postural restrictions necessary for management of posterior canal benign paroxysmal positional vertigo? Ann Otol Rhinol Laryngol. (2011) 120:460–4. doi: 10.1177/000348941112000707, PMID: 21859055

[26] Simoceli L Bittar RS Greters ME. Posture restrictions do not interfere in the results of canalith repositioning maneuver. Braz J Otorhinolaryngol. (2005) 71:55–9. doi: 10.1016/S1808-8694(15)31285-4, PMID: 16446892 PMC9443491

[27] Nuti D Nati C Passali D. Treatment of benign paroxysmal positional vertigo: no need for postmaneuver restrictions. Otolaryngol Head Neck Surg. (2000) 122:440–4. doi: 10.1067/mhn.2000.97986 10699824

[28] Moon SJ Bae SH Kim HD Kim JH Cho YB. The effect of postural restrictions in the treatment of benign paroxysmal positional vertigo. Eur Arch Otorrinolaringol. (2005) 262:408–11. doi: 10.1007/s00405-004-0836-7, PMID: 15378315

[29] Roberts RA Gans RE DeBoodt JL Lister JJ. Treatment of benign paroxysmal positional vertigo: necessity of postmaneuver patient restrictions. J Am Acad Audiol. (2005) 16:357–66. doi: 10.3766/jaaa.16.6.4, PMID: 16178407

[30] Ganança FF Simas R Ganança MM Korn GP Dorigueto RS. Is it important to restrict head movement after Epley maneuver? Braz J Otorhinolaryngol. (2005) 71:764–8. doi: 10.1016/S1808-8694(15)31246-5, PMID: 16878246 PMC9443566

[31] Fyrmpas G Rachovitsas D Haidich AB Constantinidis J Triaridis S Vital V. Are postural restrictions after an Epley maneuver unnecessary? First results of a controlled study and review of the literature. Auris Nasus Larynx. (2009) 36:637–43. doi: 10.1016/j.anl.2009.04.004, PMID: 19410397

[32] Devaiah AK Andreoli S. Postmaneuver restrictions in benign paroxysmal positional vertigo: an individual patient data meta-analysis. Otolaryngol Head Neck Surg. (2010) 142:155–9. doi: 10.1016/j.otohns.2009.09.013, PMID: 20115966

[33] Mostafa BE Youssef TA Hamad AS. The necessity of post-maneuver postural restriction in treating benign paroxysmal positional vertigo: a meta-analytic study. Eur Arch Otorrinolaringol. (2013) 270:849–52. doi: 10.1007/s00405-012-2046-z, PMID: 22588196

[34] McGinnis PQ Nebbia M Saez L Rudolph K. Retrospective comparison of outcomes for patients with benign paroxysmal positional vertigo based on length of postural restrictions. J Geriatr Phys Ther. (2009) 32:168–73. doi: 10.1519/00139143-200932040-00005, PMID: 20469566

[35] Soto-Varela A Rossi-Izquierdo M Santos-Pérez S. Can we predict the efficacy of the semont maneuver in the treatment of benign paroxysmal positional vertigo of the posterior semicircular canal? Otol Neurotol. (2011) 32:1008–11. doi: 10.1097/MAO.0b013e3182267f02, PMID: 21725255

[36] Otsuka K Suzuki M Shimizu S Konomi U Inagaki T Iimura Y. Model experiments of otoconia stability after canalith repositioning procedure of BPPV. Acta Oto Laryngologica. (2010) 130:804–9. doi: 10.3109/00016480903456318, PMID: 20095871

